# Management of Stent Thrombosis Post-Percutaneous Coronary Intervention and Associated Rare Complications

**DOI:** 10.7759/cureus.18370

**Published:** 2021-09-29

**Authors:** Kahtan Fadah, Divyank Patel, Kunal Mishra, Rakhee Makhija, Tariq Siddiqui

**Affiliations:** 1 Internal Medicine, Texas Tech University Health Sciences Center El Paso Paul L. Foster School of Medicine, El Paso, USA; 2 Cardiovascular Medicine, Texas Tech University Health Sciences Center El Paso, El Paso, USA; 3 Cardiology, Texas Tech University Health Sciences Center El Paso, El Paso, USA

**Keywords:** very late stent thrombosis, subacute stent thrombosis, thrombosis, stents, pci

## Abstract

Stent thrombosis is a devastating complication of percutaneous coronary intervention (PCI) associated with significant morbidity and mortality. Progressive technical advancements from balloon angioplasty to bare-metal stent and drug-eluting stent placement have reduced the incidence of stent thrombosis. Definitive management and preventive methods are still negligible. Here, we describe two cases of definite subacute stent thrombosis of the right coronary complicated by pericarditis and very late left anterior descending stent thrombosis after the intervention in the right coronary artery. In both cases, antiplatelet treatment with clopidogrel showed excellent compliance. Therefore, after successful PCI, we switched both cases from clopidogrel to potentially more potent antiplatelet treatment, such as ticagrelor, to reduce the occurrence of stent thrombosis in the future.

## Introduction

Percutaneous coronary intervention (PCI) is a commonly performed procedure with a high success level. Nevertheless, stent thrombosis is a serious but likely infrequent complication. Currently, stent thrombosis is classified as acute <24 hours, subacute <30 days, or late >30 days. While the most preferred approach remains to repeat PCI in all three types, thrombolytic therapy is a viable strategy in facilities without catheterization (cath) laboratories [1]. The BASKET-LATE trial showed that late stent thrombosis is more frequent compared to early and subacute thromboses, towards which guidelines have been focused [2]. Moreover, the consequences of early and subacute stent thrombosis are known to be more severe [3]. Here, we discuss two contrasting cases; one of early subacute stent thrombosis managed with urgent coronary angiography complicated by pericarditis, and another of very late in-stent thrombosis after being noted to be patent just four days prior.

## Case presentation

Case 1

**Figure 1:**
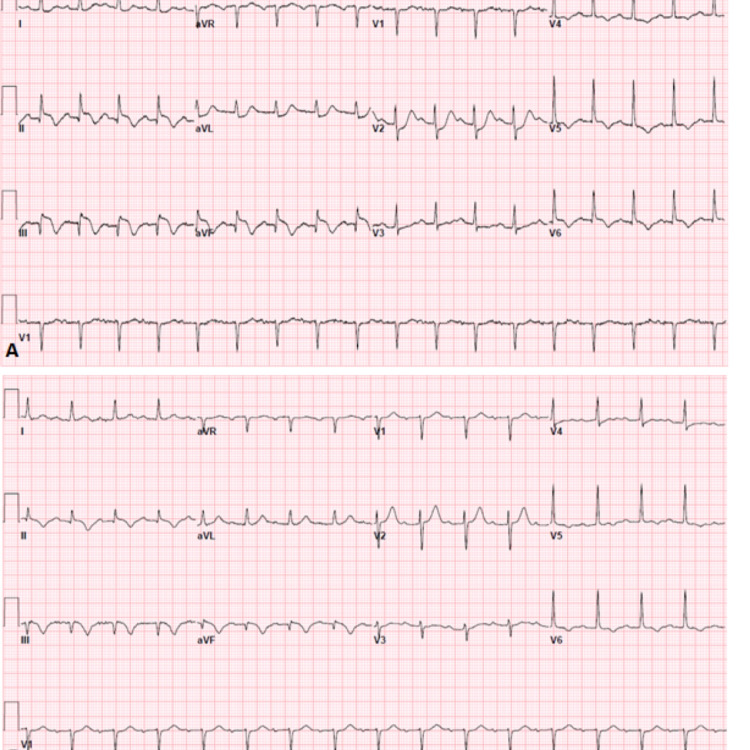
(A) Inferior-posterior STEMI, ST-elevation noted in leads II, III, and aVF. (B) Post-PCI showing evolving changes with smaller remaining ST-elevation in lead III. STEMI: ST-segment elevation myocardial infarction; PCI: percutaneous coronary intervention

A 67-year-old male with basal cell carcinoma, hypertension (HTN), and prediabetes presented initially with severe mid-sternal chest pain radiating to the right shoulder and neck. Initial electrocardiogram (ECG) (Figure 1, Panel A) showed inferior-posterior ST-segment elevation myocardial infarction (STEMI) managed in accordance with acute coronary syndrome (ACS) protocol.

**Figure 2:**
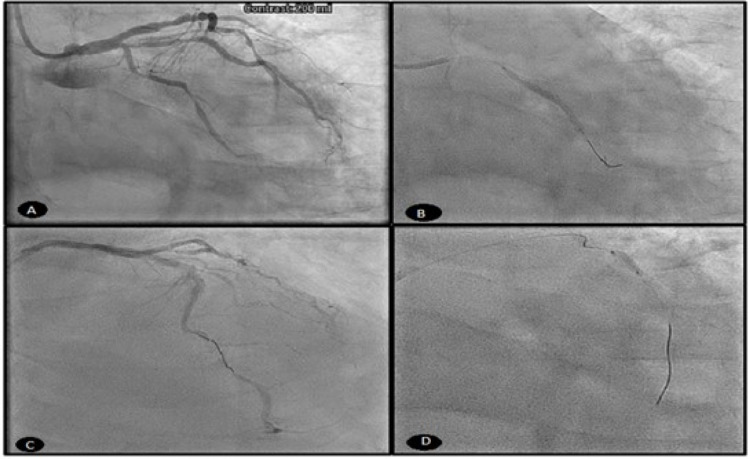
(A) Angiogram showing stenosis in the LAD. (B-D) Stent placement in the RCA, LAD, and proximal LCx. LAD: left anterior descending artery; RCA: right coronary artery; LCx: left circumflex artery

He successfully underwent emergent aspiration thrombectomy and PCI with the drug-eluting stent (DES) of mid to distal right coronary artery (RCA). The patient was noted to have stenosis of the proximal to the mid-left anterior descending artery (LAD) as well as the proximal left circumflex artery (LCx), as shown in Figures 2A-2D. Post-PCI ECG (Figure 1B) showed resolving ST-elevation in leads II, III, and aVF compared to pre-PCI ECG.

**Figure 3:**
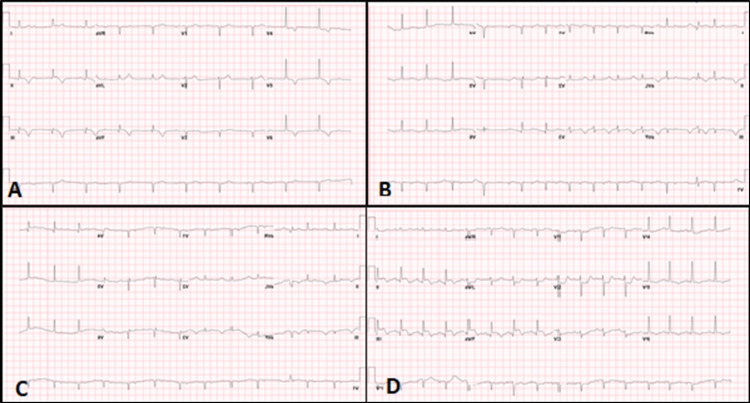
(A) Sinus rhythm with complete heart block and junctional rhythm. (B) No evidence of complete heart block, but sinus rhythm with second-degree AV block (Mobitz I) can be seen. (C) No evidence of complete heart block, but sinus rhythm with first-degree AV block with occasional premature ventricular complexes can be seen. (D) Captured three days after obtaining C revealing inferior-posterior STEMI. AV: arteriovenous; STEMI: ST-segment elevation myocardial infarction

An echocardiogram obtained at this time reported a moderate reduction of left ventricular systolic function (LVSF) with an ejection fraction (EF) of 30-35%, as well as RCA regional motion abnormality. After 24 hours post-PCI, he developed a third-degree arteriovenous (AV) block with junctional escape rhythm, as shown in Figure 3A. The patient’s vital signs were stable and did not require pacing. ECG improved to second-degree and then to first-degree AV block and subsequently to normal sinus rhythm (Figures 3B, 3C). He was hemodynamically stable thereafter and was continued on aspirin, clopidogrel, and optimized management of blood pressure.

**Figure 4:**
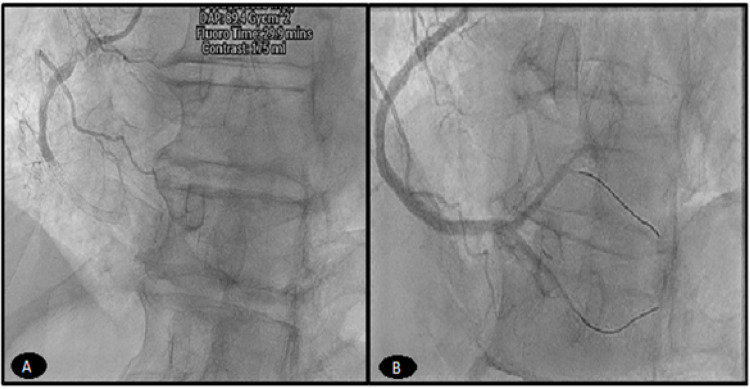
(A) RCA in-stent thrombosis developing after 72 hours. (B) Aspiration thrombectomy of mid and distal RCA stents. RCA: right coronary artery

He complained of crushing chest pain 72 hours later. ECG (Figure 3D) showed inferior-posterior infarct STEMI. The patient was taken back to the cath lab and underwent another angiogram, which showed a DES for definite early subacute stent thrombosis of RCA stents (Figure 4A). We performed aspiration thrombectomy of early subacute stent thrombosis of mid-RCA and distal RCA stents (Figure 4B). Clopidogrel resistance assay was negative at this time but we decided to switch to ticagrelor. Upon follow-up three months later, the patient had been stable and clinically improving with no further angina and continued to take medication as prescribed. Routine follow-up ECG showed normal sinus rhythm.

**Figure 5:**
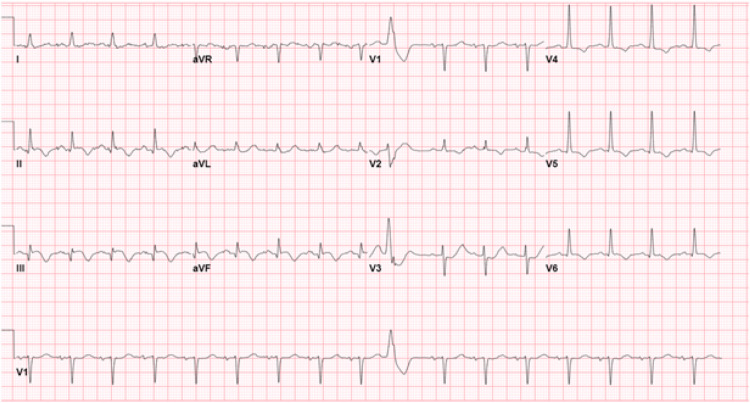
ST-elevation in leads II and III with ST-depression in V3-6.

Repeat echocardiogram three months later also revealed improved LVSF with an EF of 40-45%. However, he presented after 96 days with acute chest pain (Figure 5). Left heart catheterization was negative for thrombosis, and a diagnosis of post-ST pericarditis was made, for which he received anti-inflammatory aspirin and colchicine with subsequent improvement in pain level.

Case 2

**Figure 6:**
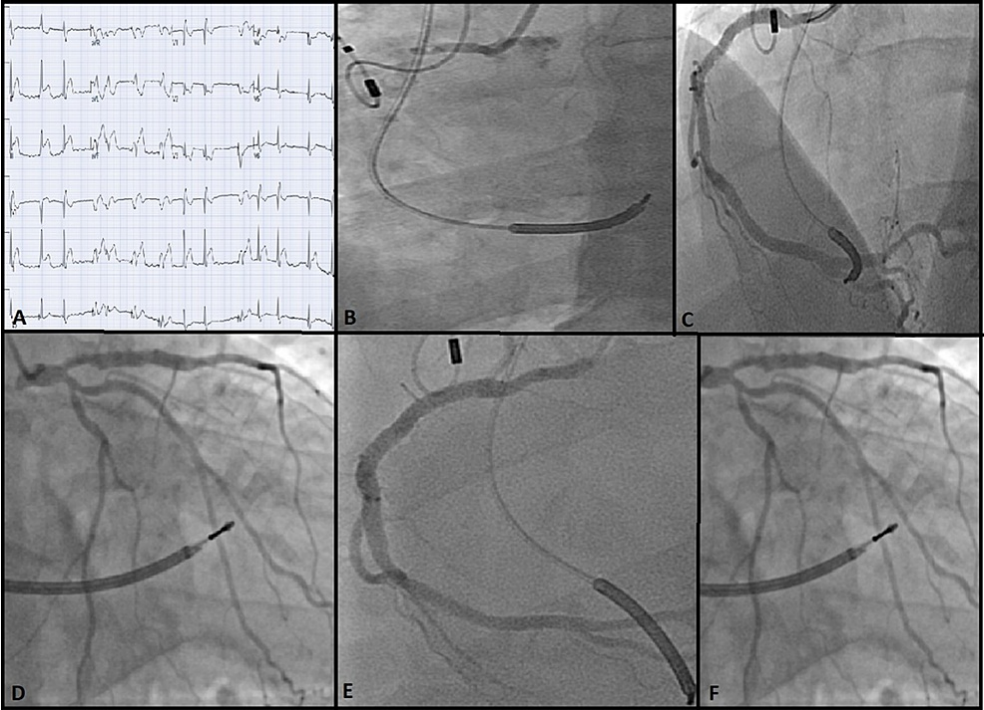
(A) Initial ECG showing inferior-posterior STEMI. (B) Angiogram of the RCA revealed acute 100% thrombotic occlusion. (C) After percutaneous intervention with DES. (D) Angiogram during index presentation revealing widely patent LAD stent. (E) Repeat angiography two days after index case demonstrating widely patent RCA. (F) Angiography at the same time as in E showing patent LAD stents. ECG: electrocardiogram; STEMI: ST-segment elevation myocardial infarction; RCA: right coronary artery; DES: drug-eluting stent; LAD: left anterior descending artery

A 51-year-old male with a LAD stent placed 10 years prior and chronic controlled atrial fibrillation on therapeutic anticoagulation with warfarin presented with typical chest pain. ECG revealed an acute inferior-posterior STEMI (Figure 6A). Emergent coronary angiography revealed proximal RCA as the culprit lesion (Figure 6B), consistent with inferior STEMI and patent LAD. Primary PCI with a 3.5 × 28 mm DES was performed and a dual antiplatelet (DAPT) regimen of aspirin 81 mg and clopidogrel 75 mg daily after a loading dose of 600 mg pre-PCI was continued (Figure 6D). An echocardiogram showed an LVEF of 30%. Repeat coronary angiogram two days later for chest pain and ventricular arrhythmia revealed patent RCA and LAD stents (Figures 6E, 6F).

**Figure 7:**
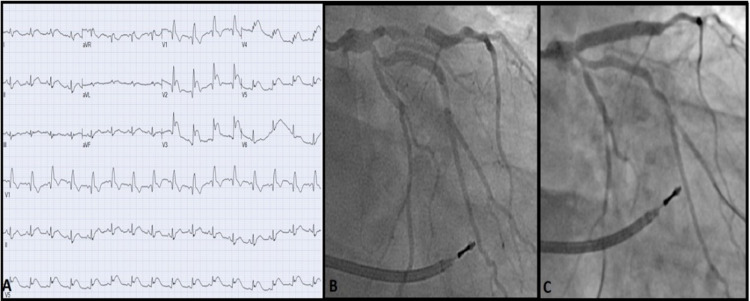
(A) Day six after the index case showing acute anteroseptal STEMI. (B) Angiogram on day six revealing definite very late in-stent thrombosis of the LAD. (C) LAD after PCI with a DES of in-stent thrombosis. STEMI: ST-segment elevation myocardial infarction; LAD: left anterior descending artery; PCI: percutaneous coronary intervention; DES: drug-eluting stent

Emergency esophagogastroduodenoscopy was performed a day later for hematemesis showing gastritis necessitating pantoprazole. Hemoglobin and hematocrit remained stable. DAPT and warfarin were continued. Four days later, chest pain recurred, and ECG showed ST-elevation in the anteroseptal leads V2-6 (Figure 7, Panel A).

Emergency coronary angiogram revealed definite very late in-stent thrombosis of the previously patent LAD stent (Figure 7, Panel B). The patient underwent primary PCI with aspiration thrombectomy and a 3.5 × 26 mm DES of LAD (Figure 7, Panel C). Clopidogrel response assay indicated normal P2Y12 receptor platelet blockade, and hypercoagulable workup was negative. Triple therapy was modified to include aspirin 81 mg, ticagrelor 90 mg BID, and apixaban 5 mg BID upon discharge.

## Discussion

PCI coupled with DAPT drastically reduces the incidence of developing stent thrombosis. Large multicenter studies previously reported a 3-4% chance of developing stent thrombosis before the adoption of high-pressure dilation, which reduced this rate to less than 1% [4,5]. In accordance with the Academic Research Consortium, stent thrombosis is categorized as acute (<24 hours), subacute (between 24 hours and 30 days), and late (extending past 30 days).

Three factors account for stent thrombosis. First, mechanical causes from turbulence and shear forces such as malposition, dissection, or oversized stent in small vessels [2]. Second, endothelial inflammation which can lead to congenital or gained hypercoagulability [6]. Third, overactivation of the platelet activation pathway causing thrombosis [7]. Implanting high-pressure deployment systems are widely used to minimize extensive interference with vessel structure around the stent site. This, in turn, decreases the proliferation of the intimal region and the chance of subacute stent thrombosis [3,8].

Late angiographic stent thrombosis is defined as thrombosis after 30 days of stent implantation. Other studies have defined “very late” as stent thrombosis after one year of implantation, which was the case in our second patient. While the incidence remains rare, late stent thrombosis has been reported in patients undergoing brachytherapy with stenting. Risk factors for late stent thrombosis include stenting across Ostia and plaque disruption. In a persistent region, extensive plaque prolapse could lead to late stent thrombosis [9]. Studies have shown that there is no difference between bare-metal stents (BMSs) and DESs in the incidence of stent thrombosis, with a cumulative incidence of 2.1-3.3% over three years [10,11]. Although early and late stent thrombosis occurs with a similar frequency after BMS or DES and outnumbers very late stent thrombosis, very late stent thrombosis has emerged as a distinct clinical entity more exclusive to (at least the first-generation) DES than BMS. Delayed healing and impaired endothelialization induced by the drug-polymer combination are the prevailing mechanisms of late and very late stent thrombosis. Cytotoxic drugs used post-PCI to inhibit smooth muscle growth simultaneously halt the endothelialization process as well. Inflammation caused by polymers used to load drugs during PCI can lead to hypersensitivity reaction by damaging vessel wall eosinophilic cells, and, hence, increasing the incidence of prothrombotic events [12].

The most practiced approach currently to manage stent thrombosis is to re-perform PCI. Our cases of subacute and very late stent thrombosis were successfully treated with aspiration thrombectomy with a high-pressure optimized stent deployment system. One of the major risks to stent thrombosis, in general, is noncompliance with DAPT. However, both patients reported compliance with clopidogrel and aspirin, and there was no reason to doubt it giving a prior long history of compliance. As recommended by the American College of Cardiology/American Heart Association guidelines, we transitioned the patients to a more potent antiplatelet ticagrelor [6,13]. The underlying application to the development of stent thrombosis in these cases remains uncertain. While PCI continues to increase worldwide, there is a tremendous need for further research into the etiology and risk factors driving stent thrombosis to determine different treatment and preventative approaches.

## Conclusions

Complications associated with stent thrombosis are rare but fatal if not managed appropriately. Repeat PCI is the leading intervention for stent thrombosis regardless of classification from acute to very late. Medication noncompliance is one of the major risk factors. However, cases of stent thrombosis in the setting of medication compliance still exist, and clinicians should consider in-stent thrombosis when patients present with new-onset chest pain. Further studies are required, particularly with the rising PCI usage globally, to determine the exact pathophysiology, treatment options, and whether more potent antiplatelet treatment offers a better chance of prevention of stent thrombosis in the long term.
